# Vitamin D Concentration in Maternal and Umbilical Cord Blood by Season

**DOI:** 10.3390/ijerph14101121

**Published:** 2017-09-26

**Authors:** Regina Wierzejska, Mirosław Jarosz, Włodzimierz Sawicki, Michał Bachanek, Magdalena Siuba-Strzelińska

**Affiliations:** 1 Department of Nutrition and Dietetics, Clinic of Metabolic Diseases and Gastroenterology, Institute of Food and Nutrition, 02-903 Warsaw, Poland; zaklad@izz.waw.pl (M.J.); msiuba@izz.waw.pl (M.S.-S.); 2 Clinic of Obstetrics, Gynecology and Oncology, 2nd Faculty of Medicine, Medical University of Warsaw, 03-242 Warsaw, Poland; ginpol@brodnowski.pl (W.S.); mbachanek@op.pl (M.B.)

**Keywords:** vitamin D, blood, pregnant women, newborn, season

## Abstract

Summer is generally considered to be the season when the body is well-supplied with vitamin D. The aim of this study was to compare maternal and umbilical cord blood concentrations of vitamin D during two extreme seasons of the year in Poland—winter and summer. A total of 100 pregnant women with no history of chronic diseases before pregnancy were included in the study. Pre-delivery maternal venous blood and neonatal cord blood samples were collected and total 25(OH)D concentration was measured. Data on vitamin D consumption (collected with the use of Food Frequency Questionnaire) and lifestyle factors were taken. Both, maternal and umbilical cord blood concentrations of vitamin D were higher in the summer group as compared to the winter group (mean 22.2 ± 6.5 ng/mL vs. 16.5 ± 8.2 ng/mL (*p* < 0.001), respectively for the mothers and 31.3 ± 9.4 ng/mL vs. 22.7 ± 11.0 ng/mL (*p* < 0.0001), respectively for the neonates). However, only 16% of the pregnant women reached the optimal vitamin D concentration during summer. Therefore, summer improves the levels of vitamin D in the body but does not guarantee the recommended concentration and supplementation throughout the whole year is essential.

## 1. Introduction

The last decade has witnessed a steady stream of reports from all over the world about vitamin D insufficiency in the general population, even in regions with high year-round sun exposure (the so-called “Mediterranean paradox”) [1,2]. The first reports on vitamin D insufficiency appeared in the 80s of the previous century but it was not until recently that the problem gained significant attention due to the growing tendency to stay indoors, avoid sun exposure, and use of sunscreen [2,3]. Vitamin D insufficiency in pregnant women increases the risk for a complicated pregnancy course and results in fetal insufficiency [4,5].

The literature offers no consensus on the recommended vitamin D concentrations or unified diagnostic criteria. Furthermore, the official nomenclature also varies (optimal, adequate, recommended, sufficient, insufficient, intermediate, suboptimal, hypovitaminosis, deficient, medium deficient, severe deficient) [5,6,7,8], which may hinder epidemiological estimates on the global scale. In light of the ongoing debate on the optimal vitamin D concentration in pregnant women, most experts are of the opinion that the 25(OH)D concentration in maternal blood should exceed 30 ng/mL [2,8,9]. However, other authors claim that maternal concentration that fully normalizes vitamin D metabolism and calcium homeostasis is at least 40 ng/mL [10], and still others that >20 ng/mL is necessary to prevent vitamin D deficiency in the newborn [11].

As dietary sources of vitamin D have failed to meet the recommended levels for years [12,13], vitamin D concentration is in fact enhanced by supplementation and exposure to sunlight [2,5]. In consequence, season of the year, geographic location and lifestyle have become the key factors as far as sources of vitamin D are concerned. The aim of the study was to evaluate maternal and umbilical cord blood vitamin D concentrations and determine the extent to which the season of the year (summer) improves these levels in Poland.

## 2. Materials and Methods

### 2.1. Study Design

The study was conducted among 100 participants at the Department of Obstetrics, Gynecology and Oncology, Medical University of Warsaw. The subjects were divided into two subgroups—group 1: “winter” (*n* = 50), including women who delivered between December and February, and group 2: “summer” (*n* = 50), consisting of women who gave birth between July and August. The pair-matching technique for the summer and winter groups was not used in our study. The sample size was determined using the mean number of women who delivered at the Clinic during one month. The study included women who presented at the hospital on weekdays, in the morning. The exclusion criteria were the following: non-Polish nationality, multiple gestation, advanced stage of the delivery, chronic maternal diseases before pregnancy, and threatened course of labor. After informed consent was obtained, maternal blood was drawn, followed by cord blood at delivery, to form a “mother-infant blood set”. The Ethics Committee of the Institute of Food and Nutrition approved of the study (Code 10/162/KB/2014).

### 2.2. Laboratory Analysis and Data Collection

Total 25(OH)D concentration was measured in the blood using immunological tests (LIAISON). The lower detection threshold for vitamin D is 4.0 ng/mL. Vitamin D consumption was evaluated on the basis of the frequency of the consumption of main dietary sources (fish, eggs, milk, and margarine —obligatorily supplemented with vitamin D in Poland), and supplements of vitamin D. A Food Frequency Questionnaire, validated at the Institute of Food and Nutrition, was used to assess the quantity of food consumed by the subjects. In order to precisely evaluate portion size, direct interviewing (face-to-face) and the “Photo Album of Meals and Products” were used for data collection. The questionnaire also included data on patient lifestyle (e.g., smoking, outdoor activity between 10:00 a.m. and 3:00 p.m., tendency to avoid the sun, use of sunscreen), weight gain during pregnancy, and sociological data. Maternal characteristics are presented in Table 1. The content of vitamin D in vitamin/mineral supplements for pregnant women, as well as single-component vitamin D preparations, was estimated on the basis of our earlier analysis [14]. The following criteria of maternal serum 25(OH)D concentration were used: recommended level >30 ng/mL, insufficiency 20–30 ng/mL, deficiency <20 ng/mL [3,6,7,8]. As for umbilical cord blood, ≥20 ng/mL was the recommended level and lower values signified vitamin D deficiency [15,16].

### 2.3. Statistical Analysis

A multivariate regression analysis was used to investigate the relationship between maternal vitamin D concentration and selected baseline characteristics (vitamin D consumption, use of dietary supplements, season, maternal age and education, gravidity, BMI, weight gain, diabetes, hypertension, smoking, use of sun screen, exposure to sun). Simple linear regression was used to analyze venous versus cord blood vitamin D concentration. After checking the normality of data distribution, adequate methods of result presentation and statistical analysis were applied. Chi-square test, Pearson’s correlation or Fisher’s exact test were used to analyze two discrete distributions. The *p*-value of <0.05 was considered as statistically significant. ROC curves were used to predict neonatal levels on the basis of maternal levels of vitamin D.

## 3. Results

### 3.1. Maternal Venous Blood by Season

**Figure 1:**
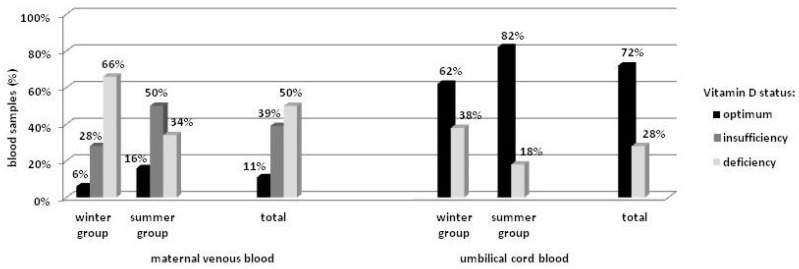
Distribution of vitamin D concentration in the blood.

Mean maternal vitamin D concentration was 19.3 ± 7.9 ng/mL, and was closely connected with the season of the year, with 16.5 ± 8.2 ng/mL for the winter and 22.2 ± 6.5 ng/mL (*p* < 0.001) for the summer group. Multivariate regression analysis revealed that mean vitamin D concentration during summer was higher by approximately 5.8 ng/mL (95% CI = [2.60, 8.93]) as compared to winter (*p* < 0.001). The season was also significantly associated with the distribution structure for vitamin D concentration (Pr = 0.005). In the winter group, deficiency (<20 ng/mL) was detected in 66% of the women, and only 6% achieved the recommended level (>30 ng/mL). In the summer group, the number of subjects with vitamin D deficiency was almost 50% lower, while the recommended level was detected in almost 3-times more subjects as compared to the winter group, but still in only 16% of the group. Out of the total, the recommended level of vitamin D concentration was found in 11% of the women (Figure 1).

### 3.2. Umbilical Cord Blood by Season

Mean umbilical cord blood vitamin D concentration was 27.0 ± 11.1 ng/mL, and was lower among neonates born in winter as compared to summer (22.7 ± 11.2 ng/mL vs. 31.3 ± 9.4 ng/mL, respectively) (*p* < 0.0001). Multivariate regression analysis revealed the mean season-related difference to be 9.6 ng/mL (95% CI = [5.31, 13.87]) (*p* < 0.0001). Similarly to the maternal values, differences in the distribution structure for vitamin D concentration between the neonatal groups were observed (Pr = 0.026). In the winter group, deficiency (<20 ng/mL) was detected in 38% of the infants, while in the summer group the number was lower by half. Overall, vitamin D deficiency was found in >25% of the neonates (Figure 1). No differences in umbilical cord blood vitamin D concentration were found with regard to gender.

### 3.3. Maternal vs. Umbilical Cord Blood

**Figure 2:**
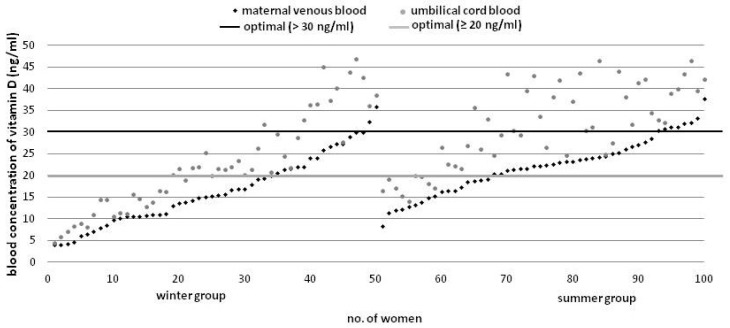
Vitamin D concentration in the mother-infant blood sets by season (ascending order).

Umbilical cord blood vitamin D concentration was higher as compared to maternal levels in all of the investigated cases (Figure 2).

**Figure 3:**
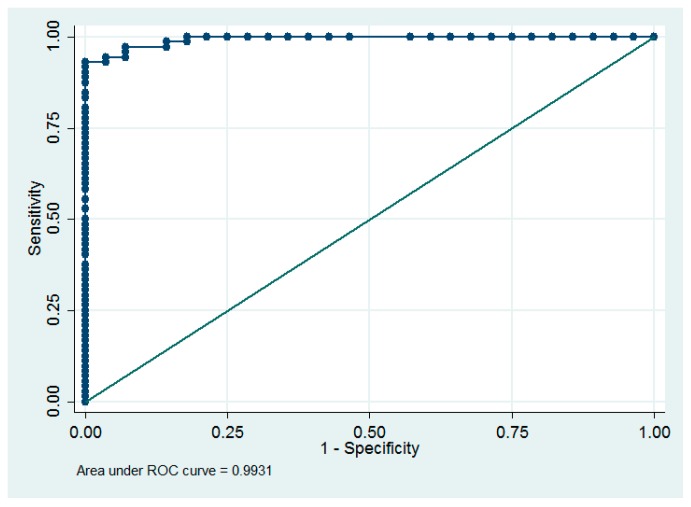
Receiver operating characteristic (ROC) curve for the dependence between maternal levels and optimal neonatal levels.

With a 1 ng/mL increase in the maternal levels, umbilical cord blood vitamin D concentration increases by approximately 1.26 ng/mL (95% CI = [1.13, 1.40] (R^2^ = 0.78, *p* < 0.0001). Additionally, we found that the diagnostic value of basing the neonatal prognosis for vitamin D concentration on the maternal levels was very high (ROC area = 0.993) (Figure 3). All neonates with cord blood vitamin D deficiency were born to mothers with <15.3 ng/mL venous blood concentration, while 67 (93%) infants with optimal levels of vitamin D were born to mothers with ≥15.3 ng/mL venous blood concentration of that vitamin, indicating that the sensitivity and specificity of the predicted normal cord blood levels were 93% and 100%, respectively at the maternal concentration of ≥15.3 ng/mL.

### 3.4. Other Factors Related to Serum Vitamin D Concentration

Multivariate regression analysis revealed that, apart from the season of the year, vitamin D concentration was affected by the following factors: maternal use of vitamin D supplements, weight gain in pregnancy, gestational diabetes mellitus (GDM), and—in case of cord blood—also with maternal age. Mean concentration among women who supplemented their diet with single-component vitamin D preparations (mean content: 25 µg) increased by 6.3 ng/mL (95% CI = [1.77, 10.84]) as compared to non-users of supplements (*p* < 0.01), while cord blood levels were higher by 12.1 ng/mL (95% CI = [6.06, 18.19]) (*p* < 0.001). No significant differences were found with regard to the use of other vitamin/mineral supplements, either in maternal (*p* = 0.28), or cord blood (*p* = 0.26). Excessive weight gain was negatively correlated with vitamin D concentration in venous and cord blood, and was lower by approximately 4.0 ng/mL (95% CI = [−7.96, −0.02]) in the mothers and by 5.7 ng/mL (95% CI = [−11.02, −0.41]) in their neonates as compared to normal weight gain (*p* < 0.05). Also, we found that vitamin D concentration was higher by approximately 5.4 ng/mL among women with GDM (95% CI = [0.39, 10.34]) as compared to healthy mothers (*p* < 0.05), which resulted in elevated levels by approximately 7.2 ng/mL (95% CI = [0.54, 13.84]) also in neonatal cord blood (*p* < 0.05). Mean vitamin D concentration in cord blood was lower by 4.8 ng/mL (95% CI = [−9.41, −0.28]) in neonates born to mothers aged <30 years as compared to ≥30 years (*p* < 0.05). Dietary intake of vitamin D (without supplementation) by pregnant women was significantly below recommendations (median 2.1 µg/day) and was not significantly associated with blood vitamin D levels. Smoking during pregnancy, use of sun screen, and gravidity were also not associated.

## 4. Discussion

In light of current recommendations, mean maternal vitamin D concentration in our study population was deficient. The summer group had higher values, which most probably had its source in more sun exposure, as there were no differences in vitamin D intake between both groups. Nevertheless, the patients were asked to estimate only the amount of time they spent outside in the summer between 10:00 a.m. and 3:00 p.m., which does not provide direct information about the exact duration of exposure to sun. Our results are consistent with the findings of other authors, who have also confirmed vitamin D deficiency among pregnant women in Poland [3,5,17,18,19], although reports in the Polish literature on better vitamin D saturation in the summer are less unambiguous. In that respect, our results are in agreement with the study by Skowrońska-Jóźwiak et al. and Zasimovich et al. who also confirmed season-dependent variability in vitamin D levels, with the highest values in summer and the lowest in winter [3,17]. However, Bartoszewicz et al. demonstrated only slight and statistically insignificant increase in their summer group [6], while Domaracki et al., reported no differences between the two seasons [18]. Reports from other European countries also found a tendency for higher vitamin D levels among pregnant women during summer, e.g., in Germany which is located at a similar latitude as Poland [20], as well as countries with very different geographic location and sun exposure, such as Greece [12], or Norway [21]. Higher maternal vitamin D concentration during summer resulted in lower rates of mothers with vitamin D deficiency as compared to the winter group (34% vs. 66%), but the scale of vitamin D insufficiency remains significant. Overall, only 11% of the subjects reached the recommended level, which is consistent with the recent study in the Polish population (10.8%) [18]. As far as vitamin D deficiency is concerned, the situation in Poland is comparable to that seen in neighboring countries. The recommended levels of vitamin D were found in only 5% of the pregnant women in Germany [20], and 16.5% in Ukraine [22]. In our study, the high number of women with vitamin D deficiency might result from avoidance of sun exposure between 10:00 a.m. and 3:00 p.m. and using sunscreen, which was reported by 46% and 26% of the women from the summer group, respectively. Baczyńska-Strzecha et al. found that adequate, according to the patients, time of sun exposure was declared by only 25% of the women from the premature labor group and 47.5% from the term group [19]. Avoidance of sun exposure has also been observed in other countries [23,24]. A study conducted in Belgium found that pregnant women who avoid sun exposure are at a 2-fold higher risk for severe vitamin D deficiency [25].

Due to the fact that weather conditions, including the increasingly common heat waves which prevent exposure to sun, remain beyond human control, supplementation seems to be the best method of preventing vitamin D insufficiency. Regardless, despite vitamin/mineral supplementation in pregnancy, maternal intake of vitamin D remains below the recommended levels, mostly because 75% of the multi-component supplements for pregnant women which are available in Poland contain small amount of vitamin D (5–10 µg) [14], as compared to the recommended dose of 37.5–50 µg [8]. Small doses of vitamin D do not meet the requirements of the body, and remain ineffective as preventive measures [23,26,27,28]. According to the European Union law, producers of dietary supplements need not comply with the recommended doses of nutrient content. Thus, their content is established entirely by the manufacturers [29]. The problem of low doses of vitamin D in preparations for pregnant women appears to be global. Lack of compliance with the contemporary recommendations has been voiced by experts in Belgium [25], Turkey [23], and Canada [30]. Only 15% of the women from this study used single-component preparations, with higher vitamin D content (mean 25 µg) [14], which automatically resulted in higher maternal and cord blood levels.

Consumption of sea fish in Poland continues to be low (approximately 15 g/day) [31]. Thus, it seems impossible for it to ever become a significant source of vitamin D, especially in light of exaggerated media reports on chemical pollutants absorbed by the fish.

In all mother-infant blood sets, umbilical cord blood vitamin D concentration was higher by 7.7 ng/mL on average (approximately 40%), as compared to maternal levels, which is consistent with most studies worldwide, claiming that cord blood level was higher than maternal venous blood level by 3.9–8.0 ng/mL [7,12,26,32]. Godand et al. [21] found a reverse significant correlation, while Halicioglu et al. [23] reported comparable concentrations in both, maternal and umbilical cord blood. In our study, we found that maternal vitamin D concentration of >15.3 ng/mL, i.e., half the recommended dose, would be sufficient to meet the demands of the developing fetus. Streym et al. declared that maternal vitamin D concentration which is necessary for the fetus ought to be >20 ng/mL [11].

In our study, we confirmed detrimental effects of excessive weight gain in pregnancy which results in diminished vitamin D concentration. Despite a lack of consensus in the literature [6,12,23,33], prevention of excessive weight gain is important enough to monitor and mention the problem during each check-up visit. Elevated levels of vitamin D in mothers with GDM remain a surprise. It is most probably the result of the fact that GDM women in this sample more often used single-component vitamin D preparations, or even several supplements at once, as compared to healthy women (35.8% vs. 11.5%, respectively). As vitamin D concentration was measured immediately before the delivery, we lack data on pre-pregnancy, as well as the first trimester of pregnancy, values. Thus, we were unable to evaluate the relationship between vitamin D levels and the development of GDM.

The absence of a correlation between blood vitamin D levels and its intake from the diet (no supplementation) is most probably a direct result of low vitamin D intake (2.1 µg/day), which is consistent with reports of other authors at a daily intake of 0.8 µg [12]. In light of the current norms in Poland [34], adequate consumption of vitamin D from dietary sources was reported by only 6% of the investigated women. It seems that the estimated vitamin D consumption is a reliable result as it is consistent with other reports, which for years have indicated a consistently low amount of vitamin D in the diet of Polish pregnant women (2.6–2.8 µg/day) [13,35]. The same has been found for young non-pregnant women, who consume on average 2.4 µg/day of vitamin D [36]. In our study, vitamin D intake was not connected with education or age (data not presented). The association between vitamin D consumption and financial status was not investigated, which is one of the limitations of our study because that factor might have affected the diet of the investigated population. Although the study was conducted in the capital city of Poland, where mean net monthly income is the highest in the country, vitamin D intake was low.

Other limitations of our study include a relatively small sample size, which was the result of the number of deliveries at the Clinic and the nature of the study, i.e., the winter-summer comparison. Also, the study was not conducted on a representative sample of Polish pregnant women but only a group of patients from one city, in the central part of Poland, thus general conclusions ought to be drawn with caution. Regardless, no significant differences in sun exposure between the north and the south of the country have ever been reported, so the effect of the geographic location and climatic factors seems to be insignificant. It is consistent with the national studies, on a representative sample, which have determined the effect of the geographic latitude on serum vitamin D concentration to be of minimal importance [37].

As for multiparity, due to small sample size we did not evaluate the relationship between parity and time elapsed between subsequent gestations, which might have affected the vitamin D status in the body. We also did not investigate pre-pregnancy vitamin D intake, which may have had some impact on its concentration in the first trimester. Also, it is vital to bear in mind that in our study the analysis of vitamin D level was conducted on the day of the delivery, which does not signify that the concentration was typical for the entire course of pregnancy or at least its significant part. Noteworthy, type and factor of the sun screen was also not specified, which might have been a confounding factor.

## 5. Conclusions

On the basis of the findings of our study, conducted among Polish pregnant women, it seems safe to conclude that significantly low levels of vitamin D concentration among pregnant women in Poland may in fact be a common occurrence. During the summer season, even despite visible improvement, a large group of women still remains at risk for vitamin D deficiency. However, the conclusions about neonates are more favorable due to both, higher umbilical cord blood levels as compared to maternal concentration and a lower criterion of normal vitamin D levels for fetuses (by 10 ng/mL). In our study, we found that the lowest maternal vitamin D level should be ≥15.3 ng/mL in order to meet the demands of the fetus. However, that level does not apply to the overall effect of vitamin D on the course of pregnancy and maternal health.

## Figures and Tables

**Table 1:** Maternal characteristics.

Parameter	Winter Group (*n* = 50)	Summer Group (*n* = 50)	*p*-Value
age (in years) mean ± SD	29.7 ± 4.4	30.3 ± 4.4	NS
education			
higher, (%)	64	68	NS
other, (%)	36	32	
gravidity			
primipara, (%)	40	44	NS
multipara, (%)	60	56	
pre-pregnancy BMI (mean) ± SD	22.4 ± 3.3	23.1 ± 3.9	NS
weight gain			
inadequate, (%)	24	28	NS
normal, (%)	34	32	NS
excessive, (%)	42	40	NS
gestational diabetes, (%)	14	8	NS
pregnancy induced hypertension, (%)	4	14	0.0500
smoking during pregnancy, (%)	14	16	NS
professionally active during pregnancy, (%)	56	60	NS
supplementation with vitamin/mineral preparations, (%)	88	90	NS
supplementation with single-component vitamin D preparations, (%)	16	14	NS
fish consumption (at least once a week), (%)	46	38	NS
daily consumption of vitamin D—diet (µg), median (min–max)	2.11 (0.24–6.58)	2.07 (0.65–11.48)	NS
number of women with adequate daily consumption of vitamin D from the diet (≥ 5 µg), (%)	6	6	NS
daily consumption of vitamin D—diet and supplements (µg), median (min–max)	14.35 (0.82–53.85)	14.07 (0.91–98.93)	NS
time outside between 10 a.m. and 3 p.m. (mean) ± SD		2 h 18 min ± 1 h 34 min	
avoiding sun exposure between 10 a.m. and 3 p.m., (%)		46	
sunscreen, (%)		26	
maternal serum vitamin D concentration, (ng/mL) mean ± SD	16.5 ± 8.2	22.2 ± 6.5	<0.001
umbilical cord blood vitamin D concentration, (ng/mL) mean ± SD	22.7 ± 11.2	31.3 ± 9.4	<0.0001
sex of the newborn			
male, (%)	54	48	
female, (%)	46	52	NS

NS—Not significant.
